# Retraction: Downgrading MELD Improves the Outcomes after Liver Transplantation in Patients with Acute-on-Chronic Hepatitis B Liver Failure

**DOI:** 10.1371/journal.pone.0220487

**Published:** 2019-07-25

**Authors:** The PLOS ONE Editors

Concerns have been raised that the transplants performed in the local context at the time of the procedures reported in this article [1] may have involved organs/tissues procured from prisoners [2].

Details as to the donor sources and methods of obtaining informed consent from donors were not reported in [1], and when following up on these concerns the authors did not clarify these issues or the cause(s) of donor death in response to journal inquiries. International ethical standards call for transparency in organ donor and transplantation programs and clear informed consent procedures including considerations to ensure that donors are not subject to coercion [3, 4, 5].

The authors stated that no vulnerable populations were involved in their research and all organs were obtained voluntarily but did not provide ethics approval documentation or consent forms to support their claim or clarify whether organs had been procured from prisoners.

The authors did not respond to inquiries about the availability of underlying data supporting this study.

Owing to the lack of documentation to demonstrate that this study had prospective ethical approval, insufficient reporting, unresolved concerns around the source of transplanted organs and whether they included organs from prisoners, and in compliance with international ethical standards for organ/tissue donation and transplantation, the *PLOS ONE* Editors retract this article.

One of the corresponding authors, Shusen Zheng, notified the journal that all authors disagree with the retraction. The other authors either could not be reached or did not respond directly.
